# Evaluating the Impact of Test-and-Treat on the HIV Epidemic among MSM in China Using a Mathematical Model

**DOI:** 10.1371/journal.pone.0126893

**Published:** 2015-06-03

**Authors:** Sitong Luo, Litao Han, Hongyan Lu, Zhi Dou, Qian Tao, Kaveh Khoshnood, Zunyou Wu, Jie Xu

**Affiliations:** 1 Division of Prevention Intervention, National Center for AIDS/STD Control and Prevention, Chinese Center for Disease Control and Prevention, Beijing, China; 2 Department of Epidemiology and Biostatistics, Peking Union Medical College, Beijing, China; 3 School of Information, Renmin University of China, Beijing, China; 4 Institute for AIDS/STD Control and Prevention, Beijing Center for Disease Control and Prevention, Beijing, China; 5 Department of Epidemiology of Microbial Diseases, Yale University, New Haven, Connecticut, United States of America; Fudan University, CHINA

## Abstract

**Background:**

Various studies have modeled the impact of test-and-treat policies on the HIV epidemics worldwide. However, few modeling studies have taken into account China’s context. To understand the potential effect of test-and-treat on the HIV epidemic among men who have sex with men (MSM) in China, we developed a mathematical model to evaluate the impact of the strategy.

**Method:**

Based on the natural history of the CD4 count of people living with HIV and AIDS (PLWHA), we constructed a dynamic compartmental model of HIV transmission among Chinese MSM to project the number of HIV new infections and prevalence over 10 years. We predicted the annual number of HIV new infections and the total number of MSM living with HIV and AIDS (based on Beijing data) between 2010 and 2022 under the following conditions: (1) current practice (testing rate of 50% and ART coverage of 39%); (2) both testing rate and ART coverage increasing to 70% in 2013; (3) both testing rate and ART coverage increasing to 90% in 2013; and (4) both testing rate and ART coverage increasing gradually every year until 90% since 2013.

**Results:**

Based on our model, if the HIV test-and-treat policy was implemented among Chinese MSM, the total number of HIV new infections over 10 years (2013-2022) would be reduced by 50.6-70.9% compared with the current policy. When ART coverage for PLWHA increased to 58% since 2013, the ‘turning point’ would occur on the curve of HIV new infections by 2015. A 25% reduction in annual number of HIV new infections by 2015 might be achieved if the testing rate increased from 50% to 70% and treatment coverage for PLWHA increased to 55% since 2013.

**Conclusion:**

Implementation of the test-and-treat strategy may significantly reduce HIV new infections among MSM in China. Great efforts need to be made to scale up HIV testing rate and ART coverage among Chinese MSM.

## Introduction

The HIV epidemic through sexual transmission has been increasing rapidly in China in recent years. Sexual contact has become the dominant transmission mode of the epidemic. Among HIV sexually infected cases, the growth of male-to-male sexual transmission is very significant [1]. The proportion of newly infected men who have sex with men (MSM) each year increased from 12.2% to 30% within just four years [2–4]. According to the National HIV/AIDS Comprehensive Response Information Management System (CRIMS) in China, in 2012, in most Chinese metropolitan or provincial capital cities, newly reported MSM cases had accounted for more than 50% of all annually reported cases and the proportion even reached 70% in some cities. The HIV prevalence among MSM has been continuously rising and by 2012 it has reached 6.3% [5]. A cohort study carried out in 8 cities in China from 2008–2010 indicated that HIV incidence reached as high as 5.7 per 100 person-years among urban MSM [6]. Containing HIV transmission among MSM is becoming the key issue for successfully controlling the HIV epidemic in urban areas in China.

Several studies have shown that the expansion of HIV testing and antiretroviral therapy (test-and-treat) policy is highly effective in controlling the HIV epidemic [7–17]. In 2000, Blower et al developed a statistical model to predict the effect of antiretroviral therapy (ART) in preventing HIV infection. The model showed that increasing the coverage of ART would significantly decrease both the AIDS-related death rate and HIV incidence [7]. In 2006, Granich et al used a mathematical model to predict the potential impact of scaling up HIV testing and ART on the HIV epidemic based on South Africa data. The results showed that the HIV prevalence through heterosexual transmission would decrease from the current level of 17% to 1% in fifty years after a universal testing and treatment policy was implemented [8]. However, some researchers believe that the assumptions underpinning these mathematical models were unrealistic and therefore these studies would exaggerate the effectiveness and benefits of the test-and-treat strategy [18–21].

There have been few studies looking at the potential impact of test-and-treat policy on the HIV epidemic among MSM in China. Since 2012, the test-and-treat strategy has been introduced and piloted among MSM in eight cities in China. We constructed a dynamic compartmental model (DCM) to predict the effect of this policy on the HIV epidemic among MSM. Currently, about 50% of MSM have received HIV testing in the last 12 months. For ART in China, CD4 count is the most commonly used indicator for ART initiation. In 2012, more than 70% of HIV infected MSM at CD4≤350/uL were receiving ART. ART is not provided at CD4>350/μL unless other criteria are met. In contrast, the pilot test-and-treat policy promotes universal testing for all MSM at risk and recommends immediate ART upon diagnosis regardless of the CD4 count. We assessed the impact of increasing the coverage of testing and ART to 70% and 90% on HIV incidence and prevalence and compared it with the current intervention policy.

## Methods

### Dynamic compartmental model of HIV infection among Chinese MSM (based on Beijing)

**Fig 1:**
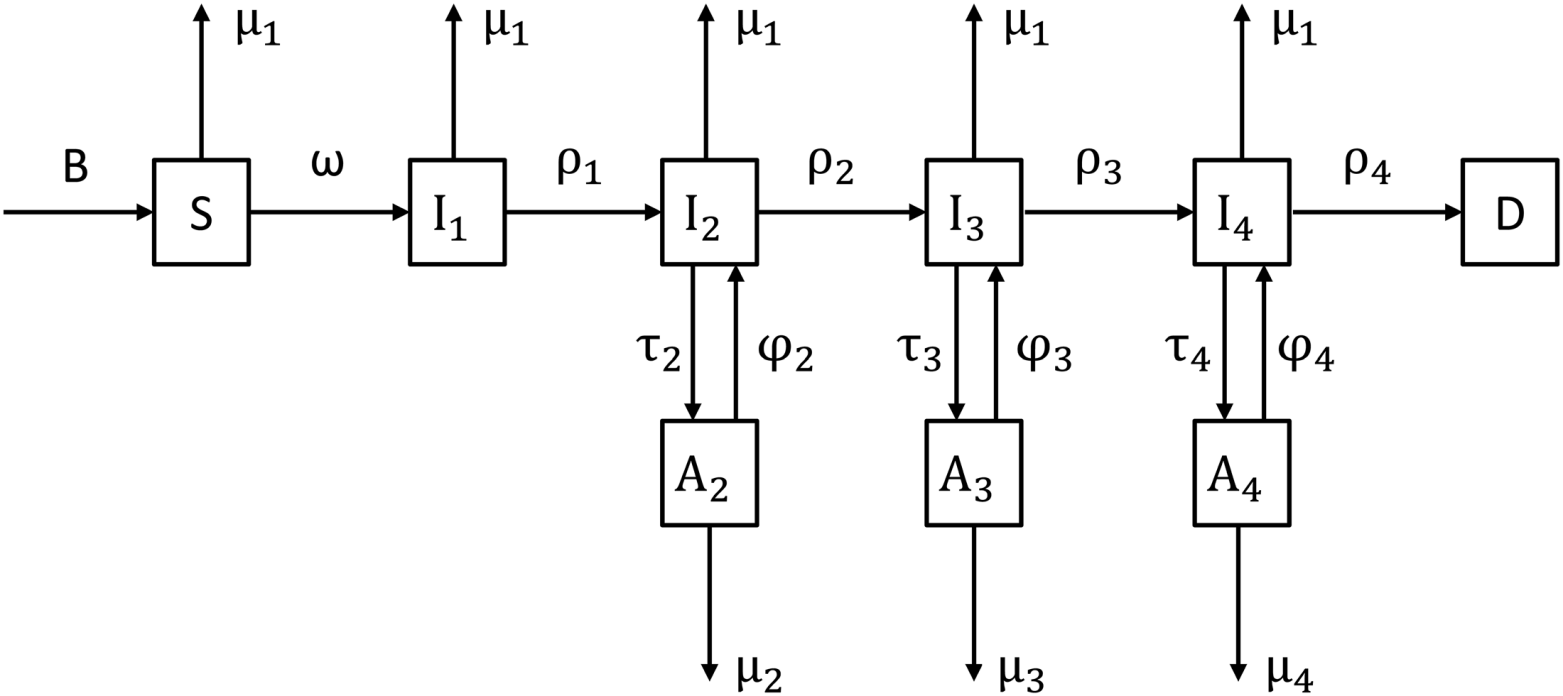
Dynamic compartmental model for HIV transmission among Chinese MSM. B represents the rate at which MSM enter into the HIV susceptible class (S). MSM infect HIV at a rate of ω, progress through four stages of HIV infection (I_1_-I_4_) at a corresponding rate ρ_i_ (i = 1–4), and then die of HIV/AIDS (D). μ_1_ represents the rate at which MSM leave the model for having no more same-sex behaviors or non-HIV related death. HIV positive MSM in each stage of HIV infection, excluding the acute stage (I_1_), are tested and put on ART at a corresponding rate τ_i_ (i = 2–4). After they receive ART, they may exit the model at a rate of μ_i_ (i = 2–4) for having no more same-sex behaviors or death. They may also stop treatment or the treatment may fail, in which case they return to the corresponding non-ART state at a rate φ_i_ (i = 2–4).

According to the natural history of CD4 count of HIV positive MSM [22–24], we constructed a dynamic compartmental model of HIV infection among Chinese MSM (Fig 1). In this DCM, we divided the MSM population into compartments according to 6 HIV infection states: (1) HIV susceptible status *S*, (2) acute HIV infection *I*_*1*_ (first 3 months of HIV infection) [22], (3) early latent infection *I*_*2*_ (CD4>350/μL), (4) late latent infection *I*_*3*_ (200/μL≤CD4≤350/μL), (5) AIDS *I*_*4*_ (CD4<200/μL), and (6) AIDS-related death *D*. We assumed that every year, there would be a number of sexually matured young men and domestic male migrants joining the MSM community (B), and some MSM might become infected with HIV because of unsafe homosexual behaviors (ω). We assumed that if the HIV infected MSM did not receive ART, they would progress through each stage of HIV infection until die from AIDS-related reasons at different rates (ρ_1_-ρ_4_), during which they might also leave the model due to non-AIDS related deaths or because they no longer practiced same-sex behaviors (μ_1_). Currently, only HIV infected MSM in late latent infection and AIDS stages may start ART (τ_3_, τ_4_) [25]. However, under the test-and-treat policy, HIV infected MSM in early latent infection stage can also be offered ART (τ_2_). We assumed that for various reasons, a proportion of MSM patients on ART might stop medication or fail in treatment (φ_2_-φ_4_), and they might also leave the model due to deaths or because they no longer practiced same-sex behaviors (μ_2_-μ_4_). In our model, we also assumed that all MSM in Beijing were uniformly mixed and that the values of parameters would not change in the whole period that we examined.

### Input Parameter

We chose the parameter values either through calculations using existing data or based on literature reviews (Table 1). The existing data included the HIV prevalence rate among MSM, CD4 count and ART uptake of HIV infected MSM which were obtained from the web-based Beijing HIV/AIDS information subsystem, part of the CRIMS. CRIMS routinely collects HIV data throughout the country including case reports, sentinel surveillance, behavioral interventions, CD4 count and ART. In our model, these data were mainly used to calculate parameters of CD4 based natural history of HIV infection with or without ART, treatment withdrawal or failure and infectiousness of HIV infected MSM. The existing data also included population data from China Population and Employment Statistics. They were used to estimate the annual number of men who newly joined the MSM population in Beijing. In case that we chose parameter values through literature, we firstly reviewed these reported values. If these values were close to each other, we chose the mean as the final parameter value. Otherwise, we defined a range covering most of the reported values to represent the parameter value. As the data in our study were anonymized and de-identified prior to analysis, informed consent was waived. The study protocol was approved by the Institutional Review Board of National Center for AIDS/STD Control and Prevention, Chinese Center for Disease Control and Prevention.

**Table 1:** Values for input parameters for the model and references.

Description of parameter	Value	References
**Demographic characteristics of MSM population**		
Proportion of MSM among sexually active men (≥15 years old)	1%-2%	[26]
Population size of MSM in 2010	108,000	^a^
Average life expectancy of HIV negative MSM	79 years	[27]
Sexually active life years of MSM	46 years	[26]
**CD4 based natural history of HIV infection with or without ART**		
Length of acute HIV infection	3 months	[22–23]
Length of early latent infection	4.33 years	[29]
Length of late latent infection	2.66 years	[29]
Length of AIDS period	2 years	[22,24]
Life expectancy of PLWHA initiating ART at early latent infection stage	79 years	[30–32]
Life expectancy of PLWHA initiating ART at late latent infection stage	33.7 years	^b^
Life expectancy of PLWHA initiating ART at AIDS stage	22.2 years	^b^
**Treatment withdrawal or failure of HIV infected MSM**		
Proportion of ART patients withdrawing or failing in treatment	3%-7%	^c^
**Infectiousness of HIV infected MSM by disease stage**		
Average number of people infected by a HIV positive MSM in a year	0.12–0.13	^d^
Acute infection	1.2–1.3	
Early latent infection	0.12–0.13	
Late latent infection	0.24–0.26	
AIDS	0.6–0.65	
**Transmission risk of HIV infected MSM on ART**		
Relative risk of Transmission of HIV infected MSM on ART versus those not on ART	0.04–0.1	[35]
**HIV testing rate and ART coverage**		
HIV testing rate under current practice	50%	^c^
HIV testing rate under test-and-treat strategy	50%-90%	
ART coverage under current practice	39%	^c^
ART coverage under test-and-treat strategy	39%-90%	

^a^ Calculated from China Population and Employment Statistics.

^b^ Calculation method described in S1 File.

^c^ Calculated from CRIMS.

^d^ Calculation method described in S2 File.

#### 1. Demographic characteristics of MSM population

We assumed that, between 2010 and 2022, the proportion of MSM among sexually active men (≥ 15 years old) in Beijing would remain at 1–2% [26]. The sexually active male population would increase every year because of the expansion of both natives and domestic immigrant population [26–28]. We assumed that the sexually active life period of MSM generally started from 18 years old and ended at 64 years old [26]. The rate at which MSM exited the model for the reason that they no longer had same-sex behaviors was calculated as the reciprocal of that duration. Population size of MSM was estimated through the calculation that the sexually active male population size times the proportion of MSM among sexually active men. We assumed that the life expectancy of HIV negative MSM in Beijing was 79 years old during 2010–2022 [27]. And the reciprocal of life expectancy was the rate at which MSM exited the model for non-HIV/AIDS death.

#### 2. CD4 based natural history of HIV infection with or without ART

We analyzed the CD4 based natural history of PLWHA with or without ART using the data in CRIMS. According to the analysis, we calculated the length of the early and late latent infection states of antiretroviral-naïve PLWHA, and obtained that the early and late latent infection lasted for about 4.33 years and 2.66 years [29]. Therefore, the rates at which antiretroviral-naïve PLWHA progressed from the early to the late latent stage and from the late latent stage to the AIDS stage were calculated as 1/4.33 and 1/2.66, respectively. We estimated the life expectancy of PLWHA on ART since initiation of treatment. For those who started ART in the late latent infection or AIDS stage, the life expectancy was 33.7 years and 22.2 years, respectively (S1 File). Similarly, the rates at which PLWHA put on ART in the late latent infection or AIDS stage exited the model due to death were calculated as 1/33.7 and 1/22.2, respectively. In addition to analyzing existing data, we made an assumption based on literatures that if PLWHA started ART in the early latent infection stage, they would have the same life expectancy as healthy people [30–32].

#### 3. Treatment withdrawal or failure of HIV infected MSM

The ART follow-up data in Beijing obtained from CRIMS from 2010 to 2012 showed that 3–7% of PLWHA on ART withdrew from the treatment or experienced treatment failure every year. So we assumed that during 2013–2022, the proportion of ART withdrawal and failure among PLWHA on ART every year was 3%-7%.

#### 4. Infectiousness of HIV infected MSM

Infectiousness of HIV infected MSM refers to the average number of MSM who may be infected by one HIV positive MSM in a year. We estimated the parameter value based on prevalence data obtained through sentinel surveillance in Beijing. While the values of other parameters of the model were fixed, we calculated the average number of people infected by an HIV positive MSM in a year as 0.12–0.13 when the predicted prevalence rates provided the best least square fit to those observed (S2 File). We assumed that the infectiousness of HIV infected MSM in the early latent infection stage was equal to 0.12–0.13. And the average values of infectiousness during acute infection, late latent infection and AIDS were defined as 10 times, 2 times and 5 times the average value of infectiousness in early latent infection stage, respectively [8, 9, 33, 34].

#### 5. Transmission risk of HIV infected MSM on ART

We assumed that after ART initiation, the average transmission probability of HIV positive MSM would decrease by 90–96% compared with HIV positive MSM not on ART [35]. That is, the relative risk of transmission of HIV positive MSM on ART was 0.04–0.1 compared with those not on ART.

#### 6. Current practice of testing and ART

We calculated the current HIV testing rate and ART coverage among MSM in Beijing based on HIV surveillance and ART data from CRIMS. In 2011, about 50% of active MSM received an HIV testing in the previous 12 months and knew their test results. By June of 2013, 78% of HIV positive MSM at CD4≤350/μL had received ART. Given that PLWHA at CD4≤350/μL upon diagnosis accounted for about half of the entire population with detected cases of HIV, we calculated that the current ART coverage was 39%. However, as some PLWHA at CD4>350/μL had also started ART, 39% was an underestimation of the actual current ART coverage.

### Analysis plan

We used MATLAB version 7.0.1 (Mathworks, Natick, MA) to carry out 1000 simulations for the model as some parameters were defined as intervals. Through simulations, we predicted the annual number of HIV new infections, total number of HIV new infections and total number of PLWHA among MSM in Beijing for the time frame 2010 to 2022 under the following conditions: (1) current practice (as reference): a testing rate of 50% and ART coverage of 39%; (2) 70% test-and-treat coverage since 2013 (optimal condition 1): both testing rate and ART coverage reaching 70% in 2013; (3) 90% test-and-treat coverage since 2013 (optimal condition 2): both testing rate and ART coverage reaching 90% in 2013; and (4) scaling up test-and-treat coverage every year until it reaches 90% (optimal condition 3): testing rate increasing by 5% every year from 50% to 90% through 2013 to 2020 and thereafter remaining at 90% till 2022; ART coverage increasing from 39% to 55% in 2013, increasing by 5% every year from 55% to 90% through 2014 to 2020 and remaining at 90% till 2022. We used the HIV prevalence rate observed in Beijing in 2010 as the initial value of HIV prevalence to run the model. Therefore, the HIV prevalence of each year from 2011 to 2022 was predicted with the model.

In addition, we predicted where the inflection point, also called ‘turning point’, occurred on the curve of HIV new infections if a specific level of test-and-treat coverage was reached. We also estimated the minimum test-and-treat coverage for achieving 25% reduction in HIV incidence among MSM in 2015 compared with 2010. The National Twelfth Five-Year Action Plan in China (the Action Plan) set the goal of reducing HIV incidence by 25% in 2015. Whether 25% reduction in HIV incidence among MSM can be achieved is critical for achieving the primary goal of the Action Plan.

### Model validation

We compared the predicted HIV prevalence rate in 2011, 2012 and 2013 with those from the sentinel surveillance to test the accuracy of the model-based predictions.

### Sensitivity analyses

We performed sensitivity analyses to assess to what extent changing the values of critical parameters affected our prediction in terms of HIV incidence. Parameters included infectiousness of HIV positive MSM (β), relative risk of transmission of HIV positive MSM on ART versus those not on ART (ε) and proportion of treatment withdrawal or failure (φ).

## Results

### Reduction of HIV incidence and prevalence using test-and-treat strategies

**Fig 2:**
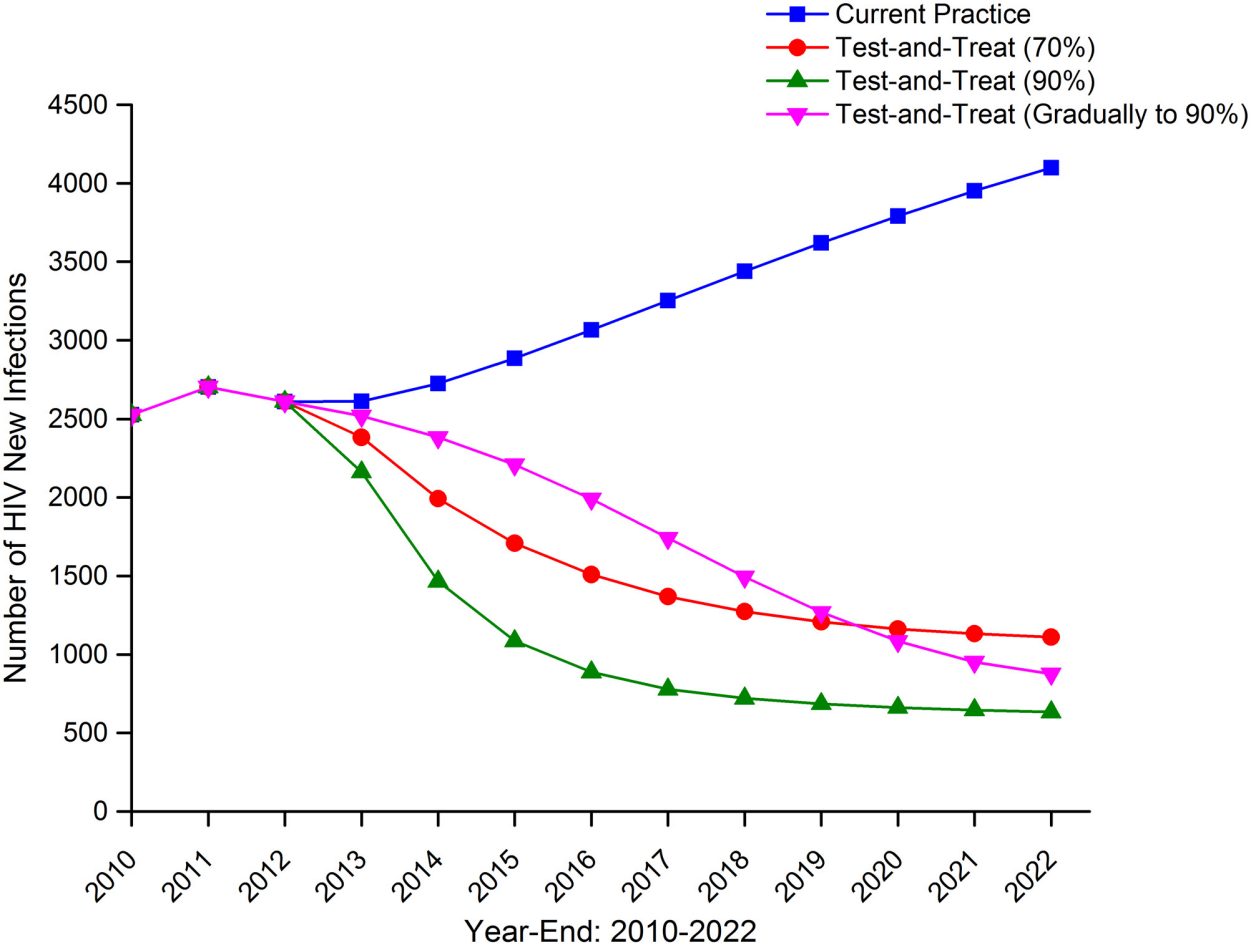
Number of HIV new infections among MSM, Beijing, 2010–2022.

**Fig 3:**
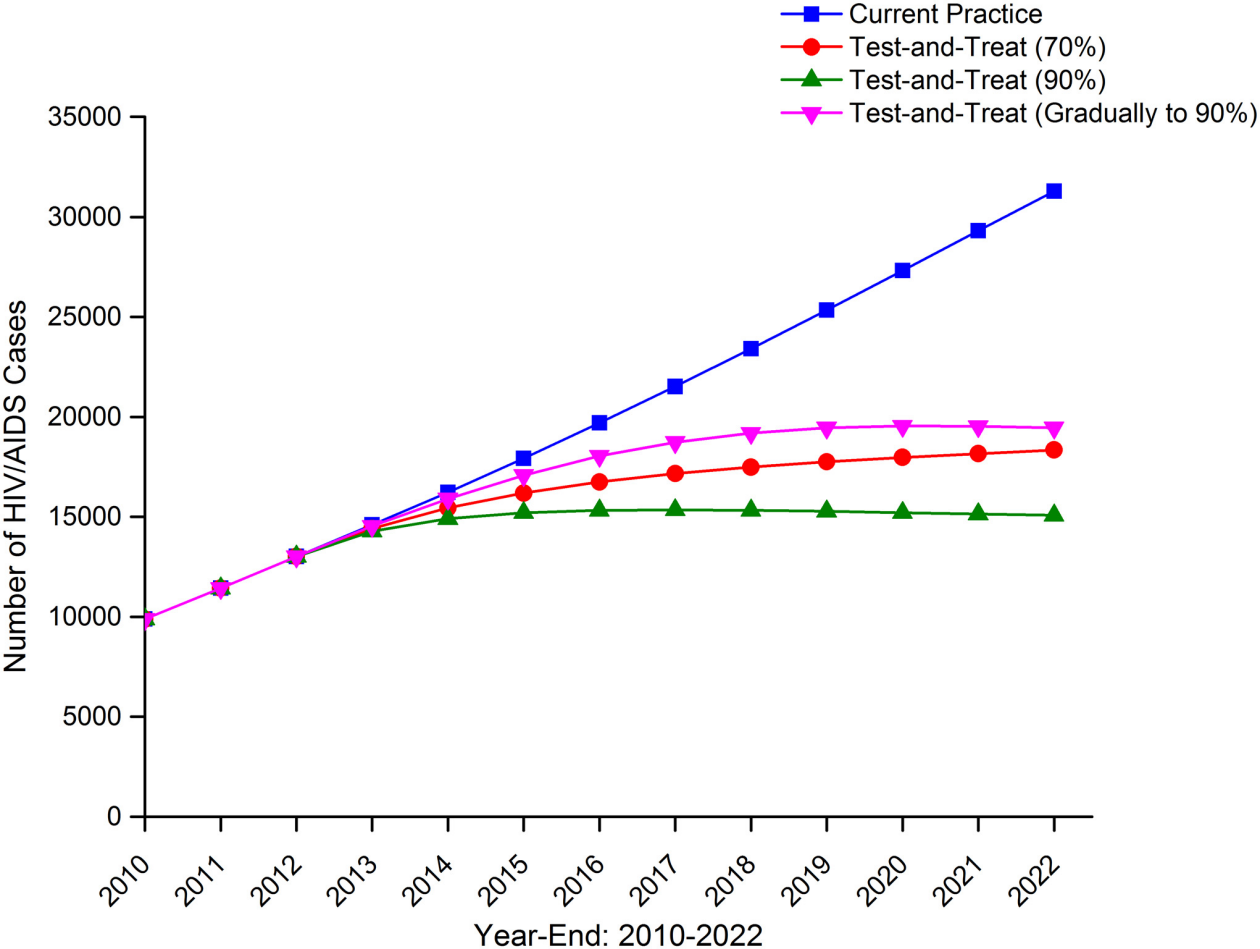
Number of MSM living with HIV/AIDS, Beijing, 2010–2022.

If the current coverage of testing (50%) and ART (39%) remained unchanged till 2022, the annual number of MSM newly infected with HIV in Beijing was expected to be increasing continuously and reach more than 4000 in 2022 (Fig 2). The total number of MSM living with HIV and AIDS would grow rapidly and have already tripled by 2022 compared with 2010 (Fig 3). If test-and-treat strategies were implemented and the coverage was increased to 70% or 90% in 2013 (optimal condition 1 and 2), the HIV incidence would begin to decrease since 2013 and reach a plateau around 2020 at a level of 500–1000 HIV new infections every year, much lower than that under current practice. If testing rate and ART coverage increased gradually every year till 90% since 2013 (optimal condition 3), HIV incidence would also begin to decrease from 2013. But in this case, the HIV incidence went down much more slowly and took longer to reach a plateau compared with increasing to 90% coverage at the beginning (optimal condition 2) (Fig 2). Under the 90% coverage of test-and-treat (optimal condition 2), the total number of PLWHA plateaued around 2015 at a level of 15000 HIV/AIDS infections. In contrast, the total number of PLWHA plateaued later at a higher level of nearly 20000 HIV/AIDS infections with 70% of test-and-treat coverage (optimal condition 1) and with gradual increasing coverage to 90% (optimal condition 3) (Fig 3).

Compared with the current policy, implementation of test-and-treat strategy would reduce the total number of HIV new infections over 10 years (2013–2022) by 55.6% and 70.9% if the coverage of HIV testing and ART increased to 70% or 90% at the beginning. If the test-and-treat coverage gradually increased to 90%, the total number of HIV new infections over 10 years would be reduced by 50.6% (Table 2).

**Table 2:** Reduction of HIV new infections among MSM under test-and-treat policies, Beijing, 2013–2022.

HIV testing rate & ART coverage	Value for parameter	Total number of new infections over 10 years (2013–2022)	% Decrease from current practice
**Current practice**			
	HIV testing rate = 50%;,ART coverage = 39%	33444	-
**Test-and-Treat**			
**Optimal condition 1**	HIV testing rate = 70%, ART coverage = 70%	14840	55.6%
**Optimal condition 2**	HIV testing rate = 90%, ART coverage = 90%	9718	70.9%
**Optimal condition 3**	Annual increase of testing rate by 5% from 50% to 90%, gradual increase of ART coverage from 39% to 90%	16511	50.6%

Note: We assume that the test-and-treat policy is started from the beginning of 2013.

### ‘Turning point’ of HIV incidence

Under the current coverage of testing and ART until 2022, the annual number of HIV new infections among MSM would rise continuously and no ‘turning point’ would occur on the curve of HIV incidence (Fig 2). If only the ART coverage increased individually, it needed to reach 55%, 58% and 60% to reverse the HIV new infection curve by 2020, 2015 and 2013, respectively. This corresponded to a highly significant increase of ART coverage for PLWHA at the early latent infection stage from less than 10% to 30%, 35% and 40%. If the HIV testing rate increased individually, it needed to get to 70% to turn the curve by 2020. When both HIV testing rate and ART coverage increased together, a moderate level of increase such as HIV testing rate from 50% to 60% and ART coverage from 39% to 50%, would allow the inflection point to occur by 2020. Increasing either of them to a higher level would reverse the curve even earlier than 2020 (Table 3).

**Table 3:** Occurrence of the ‘turning point’ on the curve of HIV incidence at different levels of test-and-treat coverage.

Occurrence of ‘turning point’	HIV testing Rate	ART coverage	ART coverage by CD4 level	
			CD4>350/uL	CD4≤350/uL
**At the end of 2020**	50%	55%	30%	80%
	55%	53%	25%	80%
	60%	50%	20%	80%
	65%	48%	15%	80%
	70%	45%	10%	80%
**At the end of 2015**	50%	58%	35%	80%
	55%	55%	30%	80%
	60%	53%	25%	80%
	65%	50%	20%	80%
	70%	48%	15%	80%
**At the end of 2013**	50%	60%	40%	80%
	55%	58%	35%	80%
	60%	55%	30%	80%
	65%	53%	25%	80%
	70%	50%	20%	80%

Note: We assume that the test-and-treat policy is started from the beginning of 2013.

### National Twelfth Five-Year Action Plan

We modeled various combinations of minimum coverage of testing and treatment by the end of 2013, to determine the conditions under which a 25% reduction in HIV new infections among MSM by 2015 might be achieved. The analysis indicated that to achieve the above goal, the ART coverage had to increase significantly. If the ART coverage increased individually, it needed to reach at least 75% corresponding to increasing ART coverage for PLWHA at early latent infection stage from less than 10% to 70%. If both HIV testing rate and ART coverage increased, a combination of HIV testing rate of 70% and ART coverage of 55% would achieve 25% reduction of HIV new infections (Table 4).

**Table 4:** Minimum test-and-treat coverage for achieving 25% reduction of HIV incidence among MSM in Beijing.

Impact on HIV incidence	HIV testing rate	ART coverage	ART coverage by CD4 level	
			CD4>350/uL	CD4≤350/uL
**25% reduction of HIV incidence among MSM in 2015 compared with 2010**	50%	75%	70%	80%
	55%	70%	60%	80%
	60%	65%	50%	80%
	65%	60%	40%	80%
	70%	55%	30%	80%

Note: We assume that the test-and-treat policy is started from the beginning of 2013.

### Model validation

According to the sentinel surveillance in Beijing, HIV prevalence rate among MSM was 7.1% (95% CI: 5.0%-9.2%), 9.5% (95% CI: 7.2%-11.9%) and 10.5% (95% CI: 8.0%-13.0%) in 2011, 2012 and 2013, respectively [36]. Our model predicted a prevalence rate of 7.1%, 8.1% and 9.2% for those years. Overall, all the predicted values fell into the 95% confidence intervals of their corresponding observed values.

### Sensitivity analyses

Sensitivity analyses indicate that the infectiousness of HIV positive MSM (both on ART and not on ART) (β) is the most sensitive parameter. If the infectiousness of HIV positive MSM (β) increased from 0.12–0.13 to 0.13–0.15, the reduction of HIV new infections over 10 years would decrease from 55.6% to 40.3% under a test-and-treat coverage of 70%. When the infectiousness decreased to 0.10–0.12, the total number of HIV new infections over 10 years would be reduced by 67.1%, much higher than 55.6% (Tables 2 and 5).

**Table 5:** Sensitivity analyses for MSM in Beijing.

Scenarios	HIV testing rate = 70%,		HIV testing rate = 90%,		Annual increase of testing rate by 5% from 50% to 90%, gradual increase of ART coverage from 39% to 90%	
	ART coverage = 70%		ART coverage = 90%			
	Total number of new infections over 10 years	% Change from current practice	Total number of new infections over 10 years	% Change from current practice	Total number of new infections over 10 years	% Change from current practice
**Current practice** ^a^	33444					
**Infectiousness of HIV+ MSM**						
0.13–0.15	19973	40.3%	12831	61.6%	22193	33.6%
0.10–0.12	11008	67.1%	7368	78.0%	12229	63.4%
**Relative risk of transmission of HIV+ MSM on ART versus those not on ART**						
0.1–0.15	17494	47.7%	12273	63.3%	19207	42.6%
0.5–0.6	40995	22.6%	36044	7.8%	43326	29.5%
**Percentage of ART withdrawal or failure**						
7%-15%	18191	45.6%	12183	63.6%	19263	42.4%
15–20%	21500	35.7%	14700	56.0%	22062	34.0%

^a^ The sensitivity analyses results are compared to estimates under current practice in which 50% of MSM receive HIV test, 39% of HIV positive MSM take ART, 12-13 per 100 people a year are infected with HIV from an HIV positive MSM, relative transmission risk of HIV positive MSM on ART is 0.04–0.1, and the proportion of ART withdrawal or failure is 3%–7%.

Relative risk of transmission of HIV positive MSM on ART (ε) and proportion of treatment withdrawal or failure (φ) were moderately sensitive. If the relative risk of transmission (ε) increased from the current level of 0.04–0.1 to 0.1–0.15, then the reduction in HIV new infections over 10 years was decreased from 70.9% to 63.3% under the 90% coverage of test-and-treat. When the relative transmission risk increased to a level as high as 0.5–0.6, there was almost no impact on reduction of HIV new infections even with 90% coverage of test-and-treat (Tables 2 and 5).

If the proportion of treatment withdrawal or failure (φ) increased from 3–7% to 7–15%, the reduction of the total number of HIV new infections over 10 years would decrease from 50.6% to 42.4% under a gradual increase to 90% coverage of test-and-treat strategy. When the proportion increased further to 15–20%, the reduction of HIV new infections over 10 years would decrease to 34.0% (Tables 2 and 5).

## Discussion

The modeling results indicate that the test-and-treat intervention can effectively reduce HIV transmission among MSM in China compared to the current practice. The total number of HIV new infections among MSM over 10 years would decline by 50.6–70.9% if improvement of test-and-treat coverage to 70% or 90% was achieved in one year or to 90% in eight years. Even under lower coverage of test-and-treat, the implementation of test-and-treat might result in HIV new infections beginning to decline, as indicated by the turning point analysis. In addition, we found that 25% reduction in HIV incidence among MSM could be achieved by 2015 with a moderate level of HIV testing rate and ART coverage such as HIV testing rate of 65% and ART coverage of 60%. Considering that HIV epidemic among MSM in China is increasing rapidly, effective reduction of HIV transmission through same-sex behaviors will be key to achieve the goal of the Chinese Twelfth Five-year Action Plan.

Many international modeling studies have also shown similar results as our study that the test-and-treat strategy could reduce HIV infection effectively. An American study found that over a 20-years period, improvements in test-and-treat practice decreased the cumulative number of new infections by a predicted 39.3% to 69.1% in an urban MSM population based on New York City [9]. Another study assessed the impact of test-and-treat strategies on HIV new infections among MSM and people who inject drugs in British Columbia, Canada, where a 37% to 62% reduction in new cases over 25 years was estimated if the proportion of eligible individuals who received ART increased from 50% to 100% [10]. A modelling study based on the MSM population in San Francisco estimated that test-and-treat could reduce 76% of HIV new infections in 5 years [11].

We compared the estimated HIV prevalence in 2011, 2012 and 2013 with the corresponding surveillance prevalence data. We found that the surveillance HIV prevalence was slightly higher than the prevalence estimated by the model. However, the two sets of results were quite close to each other, suggesting a relatively good fit of the model.

The sensitivity analysis showed that infectiousness of HIV positive MSM needed to be properly controlled. In our model, a slight increase in infectiousness of HIV positive MSM would cause a significant decrease in effectiveness of test-and-treat in reducing HIV new infections. The analysis emphasizes the importance of promoting safe sex for HIV positive MSM to avoid ‘risk compensation’ in the context of a test-and-treat strategy.

Our model was a deterministic model which overlooked the change of values of the parameters over time. Our analysis was based on data from Beijing which had rich resource of data in good quality for modeling, relatively large MSM population size and good representativeness of urban areas. However, results derived from Beijing may not be accurate for other parts of China given that China is a huge and complex country and MSM population characteristics and HIV epidemics in each region are different. For all of the calculations and estimates of model parameters, it was likely to have variations by geography. In addition, estimation of infectiousness of HIV positive MSM was based on HIV prevalence sentinel surveillance data. The sentinel surveillance employs convenient sampling approaches such as snow-ball to recruit MSM participants, which unavoidably introduces some bias into prevalence rate survey. The biased prevalence rate data might affect estimation of infectiousness of HIV positive MSM and modeling outputs.

Our modeling study indicates that implementation of the test-and-treat policy will greatly impact the HIV epidemics among Chinese MSM, and HIV epidemic can be reversed if we achieve a reasonable coverage of HIV testing and ART among MSM. The 8-city based pilot research program needs to be continued to assess the effectiveness of test-and-treat strategy in reducing HIV incidence among MSM in practice. In our model, we assumed that the condom use rate among MSM did not change. It indicates that while the test-and-treat strategy is given more priority for implementation, we need to continuously strengthen condom promotion efforts among MSM to avoid ‘risk compensation’.

## Supporting Information

S1 File Calculation method of the life expectancy of PLWHA offered ART in the late latent infection stage and AIDS stage. Technical appendix to accompany ‘‘Evaluating the Impact of Test-and-Treat on the HIV Epidemic among MSM in China Using a Mathematical Model.”
(DOC)

S2 File Calculation method of the infectiousness of HIV infected MSM. Technical appendix to accompany ‘‘Evaluating the Impact of Test-and-Treat on the HIV Epidemic among MSM in China Using a Mathematical Model.”
(DOC)
