# Supplementary material for: Evaluating the Impact of Test-and-Treat on the HIV Epidemic among MSM in China Using a Mathematical Model
Source: PLoS One. 2015 Jun 3;10(6):e0126893. doi: 10.1371/journal.pone.0126893 (PMC4454496; doi:10.1371/journal.pone.0126893)
Supplement: S1 File — Technical appendix to accompany ‘‘Evaluating the Impact of Test-and-Treat on the HIV Epidemic among MSM in China Using a Mathematical Model.” (DOC) [file pone.0126893.s001.doc]

**Calculation method of the life expectancy of PLHIV offered ART in the late latent infection stage and AIDS stage**

We used the conditional probability formula and the least squares method to estimate the survival probability distributions and expectancy of MSM infected with HIV who initiated ART at 200 ≤ CD4 ≤ 350/uL and at CD4 ≤ 200/uL based on the ART follow-up data of Beijing.

We assumed that the survival time of people infected with HIV after ART satisfied the Weibull distribution, and had a distribution function of$f=e^{-\alpha(t^{\beta})}$. Due to the variation in the values of α and β across different populations at different ART initiation times, we used available data for the specific population to determine the values of α and β and the survival expectancy of the corresponding people infected with HIV after treatment.

We let the total available number of HIV infected people who are in treatment so far be N_0_, and the longest treatment time be T.

We let M_1_, M_2_ … M_T_ be the number of people still surviving who had received ART for one, two … T years.

We let L_0_, L_1_ …L_T-1_ be the available number of people surviving who have started treatment for more than T-1 years but less than T years.

According to the Weibull distribution, the theoretical number of people who have received treatment for a year and are still alive is$N_{0}*e^{-\alpha(1^{\beta})}$. However, from the available data, we know the number of people surviving who received ART for one year is M_1_. And by the conditional probability formula, we can also predict that for people who are alive and who have received ART for less than one year until present, some will survive after one year of treatment and this number is$L_{0}*e^{-\alpha(1^{\beta}-0^{\beta})}$. Therefore, the total actual number of living people whose treatment has lasted for one year is $M_{1}+L_{0}*e^{-\alpha(1^{\beta}-0^{\beta})}$ which has a difference $\Delta_{1}=\left|M_{1}+L_{0}*e^{-\alpha(1^{\beta}-0^{\beta})}-N_{0}*e^{-\alpha(1^{\beta})}\right|$ with the theoretical number.

Similarly, the theoretical number of people who have received treatment for two years and are still alive is$N_{0}*e^{-\alpha(2^{\beta})}$. But from the available data, the number of surviving people who received ART for two years is M_2_, and we can also predict by the conditional probability formula that for people who have received ART for more than one year but less than two years, some will survive after two years of treatment, and this number is$L_{1}*e^{-\alpha(2^{\beta}-1^{\beta})}$. So, the total actual number of living people whose treatment lasted for two years is $M_{2}+L_{1}*e^{-\alpha(2^{\beta}-1^{\beta})}$ which has a difference $\Delta_{2}=\left|M_{2}+L_{1}*e^{-\alpha(2^{\beta}-1^{\beta})}-N_{0}*e^{-\alpha(2^{\beta})}\right|$ with the theoretical number.

The remaining calculations can be done in the same manner until the longest treatment time T. Finally, we can get T errors ∆_1_, ∆_2_, ···∆_T_. We used the MATLAB version 7.0.1 (Mathworks, Natick, MA) to determine the values of α and β that minimize the sum of squares of the T errors by the least squares method, and then obtained the distribution function of$f=e^{-\alpha(t^{\beta})}$. We can then estimate the survival time of people infected with HIV at different ART initiation time from the function.

The results show that if patients started ART in the late latent infection stage (200 ≤ CD4 ≤ 350/uL) or AIDS stage (CD4 ≤ 200/uL), the life expectancy after ART would be 33.7 years and 22.2 years, respectively.
