# Supplementary material for: Evaluating the Impact of Test-and-Treat on the HIV Epidemic among MSM in China Using a Mathematical Model
Source: PLoS One. 2015 Jun 3;10(6):e0126893. doi: 10.1371/journal.pone.0126893 (PMC4454496; doi:10.1371/journal.pone.0126893)
Supplement: S2 File — Technical appendix to accompany ‘‘Evaluating the Impact of Test-and-Treat on the HIV Epidemic among MSM in China Using a Mathematical Model.” (DOC) [file pone.0126893.s002.doc]

**Calculation method of the infectiousness of HIV infected MSM**

We used the mathematical backward induction method to estimate the infectiousness of HIV infected MSM which is the average number of people who may be infected by an HIV positive MSM in a year (β). The principle of this method is estimating the value of β so that there is a minimal difference between the HIV prevalence rate predicted by our model and that obtained by sentinel surveillance using the least square method, while the values of the other parameters of the model are unchanged. The HIV prevalence rates among Beijing MSM from 2011 to 2013 obtained by sentinel surveillance and the values of the other parameters of the model are listed in the table S2. We used the MATLAB version 7.0.1 (Mathworks, Natick, MA) to carry out 1000 simulations to determine the value of β that minimizes the sum of squares of the difference between the HIV prevalence rates from 2011 to 2013 predicted by our model and those obtained by the sentinel surveillance, and found a result of 0.12-0.13.

**Table S2** Values for input parameters for backward induction method

| **Description of Parameter** | **Value** | **References** |
| --- | --- | --- |
| **HIV prevalence rate** |  |  |
| 2011 | 7.1% (95% CI: 5.0%-9.2%) | [36] |
| 2012 | 9.5% (95% CI: 7.2%-11.9%) | [36] |
| 2013 | 10.5% (95% CI: 8.0%-13.0%) | [36] |
| **Demographic characteristics of MSM population** |  |  |
| Proportion of MSM among sexually active men (≥15 years old) | 1%-2% | [26] |
| Population size of MSM in 2010 | 108,000 | a |
| Average life expectancy of HIV negative MSM | 79 years | [27] |
| Sexually active life years of MSM | 46 years | [26] |
| **CD4 based natural history of HIV infection with or without ART** |  |  |
| Length of acute HIV infection | 3 months | [22-23] |
| Length of early latent infection | 4.33 years | [29] |
| Length of late latent infection | 2.66 years | [29] |
| Length of AIDS period | 2 years | [22,24] |
| Life expectancy of PLHIV offered ART from the early latent infection | 79 years | [30-32] |
| Life expectancy of patients offered ART from the late latent infection | 33.7 years | b |
| Life expectancy of patients offered ART from the AIDS stage | 22.2 years | b |
| **Treatment withdrawal or failure of HIV infected MSM** |  |  |
| Proportion of ART patients withdrawing or failing in treatment | 3%-7% | c |
| **Transmission risk of HIV infected MSM on ART** |  |  |
| Relative risk of Transmission of HIV infected MSM on ART versus those not on ART | 0.04-0.1 | [35] |
| **HIV testing rate and ART coverage** |  |  |
| HIV testing rate | 50% | c |
| ART coverage | 39% | c |

^a^ Calculated from the China population and employment statistics

^b^ Calculation method described in Supporting Information File S1

^c^ Calculated from the CRIMS
